# Are In-the-Moment Resilience Processes Predicted by Questionnaire-Based Measures of Resilience?

**DOI:** 10.1177/10731911241234220

**Published:** 2024-03-08

**Authors:** Daniel Ventus, Patrik Söderberg

**Affiliations:** 1 Åbo Akademi University, Finland

**Keywords:** resilience, stress reactivity, experience sampling, dynamic structural equation modeling

## Abstract

Research on resilience is a growing field, and resilience has been conceptualized and operationalized in multiple ways. The aim of this study was to compare the Brief Resilient Coping Scale (BRCS), a conventional measure of resilience, with within-person process indicators derived from experience sampling method (ESM). A sample of 177 teachers from southern Finland participated in the study, commencing with a startup session followed by an 8-day ESM period. Through twice-daily prompts, participants reported their immediate positive and negative affect as well as recent stressors encountered, such as workload and challenging social interactions. As expected, within-person variation in affect was predicted by stressors. However, contrary to expectations, individual differences in affective reactivity to stressors were not predicted by BRCS (β_positive affect_ [95% CI] = −.20, [−.51, .11]; β_negative affect_ = .33, [−.07, .69]). Item response theory analyses of the BRCS revealed problems with precision. The results call into question the validity of measuring resilience using single administrations of retrospective self-report questionnaires such as the BRCS.

Resilience is a concept that has been defined and measured in numerous ways. According to Luthar et al. (2000), resilience refers to “a dynamic process encompassing positive adaptation within the context of significant adversity.” Two essential aspects of this definition are (a) exposure to significant threats or adversity, and (b) positive adjustment despite major assaults on the developmental process (Luthar et al., 2000). Historically, resilience has been studied from a developmental perspective, focusing on how severe adversities such as growing up in an abusive household affect developmental trajectories, and how these trajectories are affected by risk and protective factors on an individual, community, and societal level. On the individual level, several instruments have been developed to measure individual differences in resilience, such as the Brief Resilient Coping Scale (BRCS) (Sinclair & Wallston, 2004). This retrospective self-report questionnaire asks the respondent to rate their tendency to cope with stressful situations in an adaptive and flexible way.

Recently, a growing number of scientists have directed interest toward analyzing resilience on an intraindividual process level (Kalisch et al., 2017; Ong & Leger, 2022). These authors argue that since resilience refers to a dynamic system’s ability to adapt to stressful situations, measures of resilience should analyze the resilient process as it unfolds over time. Advances in technical and statistical methods now enable for example collection of intensive longitudinal data using smartphone applications and analyzing the data using dynamic structural equation modeling (DSEM) (Hamaker et al., 2018; Hamaker & Wichers, 2017). In one operationalization, resilience is measured indirectly by analyzing affective *reactivity* to daily stressors (Ong & Leger, 2022). In highly resilient respondents, it is expected that the affective state is stable, even in the face of stressful events. Conversely, respondents with lower resilience will display higher affective reactivity. A second aspect of resilience is the *recovery* from stressors. For example, in respondents with low resilience, experiencing stressful events one day is expected to lead to increased negative affect (NA) not only on the same day but also on the subsequent day. Proponents of these methods argue that they offer increased ecological validity since questions are answered in the respondent’s natural environment, reduced recall bias since respondents give their answers in real time, and reduced social desirability bias since resilience is measured indirectly rather than as a direct self-evaluation (Myin-Germeys & Kuppens, 2021).

In the educational setting, teachers encounter many situations that generate stress, including school context challenges such as disruptive students, and disorganized leadership, as well as professional work challenges such as heavy workload and lack of time (Beltman et al., 2011). Teacher resilience has been seen not only as managing difficulties but as successful adaption despite obstacles (Howard & Johnson, 2004) and the ability of individuals to bounce back (Malloy & Allen, 2007). According to Beltman (2020) and Mansfield et al. (2016), teacher resilience is not so much a trait-like personal quality, as it is the process through which individual and contextual factors interact to overcome adversity and lead toward positive adaptation.

As can be seen, the conceptualization and measurement of resilience can differ in significant ways with regards to adversity severity, focusing on long-term development or in-the-moment reactivity, seeing resilience more as a personality trait or a dynamic reactivity at a certain point in time, and relying on retrospective cognitively reflected self-image or empirical data in the moment. Given that research on psychological resilience has proliferated during the last two decades (Su et al., 2023, Figure 1), it is imperative for the field to compare and contrast different operationalizations of the concept of resilience. For the current study, the main research question is as follows: how well are individual differences in affective reactivity to daily stressors captured by a cross-sectional measure of resilience?

**Figure 1.**
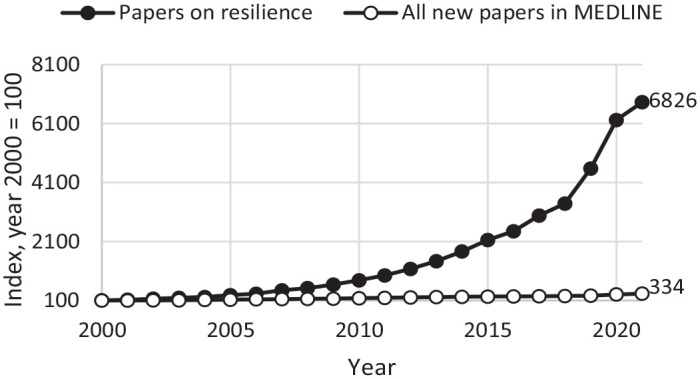
Relative Increase in Number of Papers on Resilience and Total Number of Papers Indexed in MEDLINE Yearly, During 2000–2021. *Note.* Data source for resilience trend: MEDLINE search. Data source for all new papers: Alexandru Dan Corlan. Medline trend: automated yearly statistics of PubMed results for any query, 2004. Web resource at URL: http://dan.corlan.net/medline-trend.html. Accessed: 2023-03-08.

## Aim and Hypothesis

The aim of the current study was to directly compare two measures of resilience within a single sample: the first measure being a cross-sectional questionnaire, and the second a measure of within-person processes using DSEM based on intensive longitudinal data. Based on the theoretical rationale of the BRCS measure, we tested the hypothesis that the cross-sectional measure would explain a substantial part of individual differences in affective reactivity to stressors.

## Methods

### Sample and Participants

The study was conducted in spring of 2021 in collaboration between the Faculty of Education and Welfare Studies at Åbo Akademi University, the Organization of Swedish-Speaking Teachers in Finland, and the municipalities Turku, Helsinki, and Raasepori in southern Finland. The study was conducted in accordance with the guidelines of the Finnish National Board on Research Integrity TENK (2019). According to the criteria set by the National Board, the current study did not require a review of an ethical board. Data was collected by multistage sampling. In the first stage, the project was discussed with heads of education and school principals at Swedish-speaking primary, middle, and secondary schools in the region. Out of 33 available schools, 19 chose to participate in the study (12 primary schools, 3 middle schools, and 4 secondary schools), which involved a startup session followed by an 8-day momentary assessment period. School principals decided on which weeks to conduct the study and helped to organize online startup sessions during staff meetings. In the second stage, teachers at participating schools were informed about the purpose and design of the study, in brief by email and more fully at staff meetings, including details on assessment scheduling, data management, project timeline, the voluntary nature of the study, and that the study was supported by both the Organization of Swedish-Speaking Teachers in Finland and municipality heads of education. Following a Q&A, those who opted to participate downloaded an experience sampling app (RealLifeExp by LifeData), provided in-app consent for data collection in accordance with General Data Protection Regulation (GDPR) standards, and completed a 10 to 15-minute startup survey.

Based on model requirements suggested by simulation studies by Schultzberg and Muthén (2018), we aimed for a sample size of 150 teachers (*N*) and about 20 measurement points (T); however, after discussions with participant representatives on potential participant fatigue, we settled for two measurement points during school hours each day, for a total of 16 measurement points. At startup sessions, 198 teachers downloaded our app and completed a background survey. Twenty-one cases that had less than two observations of daily stressors were removed due to the requirements of the statistical procedures. In total, 177 teachers (79% women, 19% men, 1% other or missing; mean age 42 [range 23–66]; 62% at primary school, 18% at middle school, and 30% at secondary school) completed a background survey and provided at least two momentary assessments, with an average compliance rate of 72% for momentary assessment sessions, and a total of 1,853 measurement points.

### Measures

To assess resilience in terms of a stable trait, the startup survey included the BRCS by Sinclair and Wallston (2004). The scale consists of four items designed to capture tendencies to cope with stress in adaptive manners, such as “Regardless of what happens to me, I believe I can control my reaction to it,” and participants were asked to reply to the statements on a 5-point Likert-type scale (1 = no, not at all, 5 = yes, absolutely). The four items were combined in a latent factor for analyses.

To assess momentary NA, the study used two items informed by the PANAS framework (see Watson et al., 1988). Participants were asked twice a day to assess to what extent they felt stressed, and tired, on a 5-point Likert-type scale. The items were positively correlated (*r* = .56, *p* < .001), and were added together to form a sum score for analyses. Similarly, to assess positive affect (PA), participants were asked twice a day to report to what extent they currently felt that their work was enjoyable, meaningful, and manageable, and to what extent they felt appreciated. The four items were combined in a latent factor for analyses.

To assess stressors at work, a 6-point sum score was created based on four dichotomous items on workload (e.g., “In the last few hours, there have been unexpected tasks,” “In the last few hours, I’ve had time for breaks and recreation” [reversed]), and two dichotomous items on social stress (“In the last few hours, I’ve been treated badly,” “In the last few hours, I’ve seen someone else being treated badly”).

In addition to subject-level variables from the startup session and momentary assessment response variables, the data set also included automatically generated design-related variables (subject ID and GDPR consent) and time-related variables such as day number, session number within days, response lapse (time from session notification to response initiation), and response time (time from session initiation to completion).

### Procedure

For data collection, we used the app RealLifeExp by LifeData (https://www.lifedatacorp.com/). The app operates on both Android and iOS. Study participants first downloaded the app, and then within the app used a study-specific password to download a so-called Lifepak. Once downloaded, users were informed in-app about study purpose and data management and required to provide consent before moving on to the startup session. Once the startup session is completed, the Lifepak operates locally and sends notification-initiated sessions even without an ongoing online connection. The app provides opportunities for both registered and anonymous data collection. While registered participation would be convenient for a burst-wave design, our current study only aimed for one wave of data collection and thus prioritized an anonymous approach where participants were not required to create any user login. In line with this, the GPS feature of the app was disabled centrally upon creation of the Lifepak so that the project did not collect geographical data about participants.

Within a week of the startup session, the momentary assessment period began. To keep the participant burden low and the ecological validity high, data collection was scheduled for eight working days (i.e., from Wednesday to the following Friday, with the weekend free). The study used a time-contingent sampling scheme with four assessment sessions each day: once in the morning (at 7:15), twice during school hours (at 10:15 and 13:00), and once in the evening (at 17:00). Workload, social interaction, and affect items were identical over school hour sessions, with other questions (not explored in this study) at the morning and evening sessions. Each session was initiated by a push notification from RealLifeExp, with a response window of 90 minutes to ensure that teachers would have at least one break between classes during which they would be able to respond. Participants were informed that assessment notifications would continue regardless of whether the previous assessment was completed or not.

As noted by Eisele et al. (2022), participants tend to perceive longer sessions to be more burdensome than the number of sessions, and research should therefore ensure that individual sessions are kept short. Median response lapse from notification to initiated reply in our study was 18 minutes, and median response time for sessions was 49 seconds. As an incentive to participate, each participant received monetary compensation in the form of a gift card of 20€. Participants could also opt to take part in a post-study interview on their user experiences, and 15 teachers chose to do so. In these interviews, the participants highlighted how the momentary assessments were comparatively easy and felt more meaningful to respond to than traditional surveys, but also noted that despite the typical response time being less than a minute, they sometimes did not have time for the assessments during busy school days (for more details on the interviews, see https://blogs2.abo.fi/reboot/publikationer/).

After the data collection was completed, all data were downloaded, anonymized, and stored in password-locked servers at Åbo Akademi University, with an offline external backup securely stored at the PI’s office.

### Statistical Analyses

In preparation for the analysis, startup and momentary assessment data were combined, and ordered in a long format, so that each row of data corresponded to a particular assessment moment for a given subject yet also contains all variables from the startup session. Design- and time-related variables were screened for missing data (none was found), and based on day and session variables, a time variable was computed so that each day was divided into eight 3-hour blocks (for a total of 80 blocks), and each session attached to a specific block.

To check whether the data required a multilevel approach, intraclass correlation coefficients (ICC) were calculated for dependent and independent variables by dividing between-person variance by the overall variance (between- plus within-person variance), for the latent factor PA as well as for the sum-score variables NA and stressors. Furthermore, individual panel plots were created in SPSS (version 28) by means of syntax provided by Laurenceau and Bolger (2021) and visually inspected to ensure that there were no out-of-range values or other data anomalies.

Properties of the BRCS measure were analyzed using item response theory analyses (IRT; Anderson & Miller, 2020). Assumptions of unidimensionality and local independence were assessed using exploratory and confirmatory factor analysis, respectively. For the IRT analyses, a generalized partial credit model (Muraki, 1997) was fit. The model was compared to a nested model where item discriminations were held equal. Item information curves and estimated scores on the latent resilience factor were inspected.

The main analyses were conducted within the DSEM framework (Hamaker et al., 2018), using Mplus (version 8.8). Model parameters were estimated by Bayesian estimators with non-informative priors.

**Figure 2.**
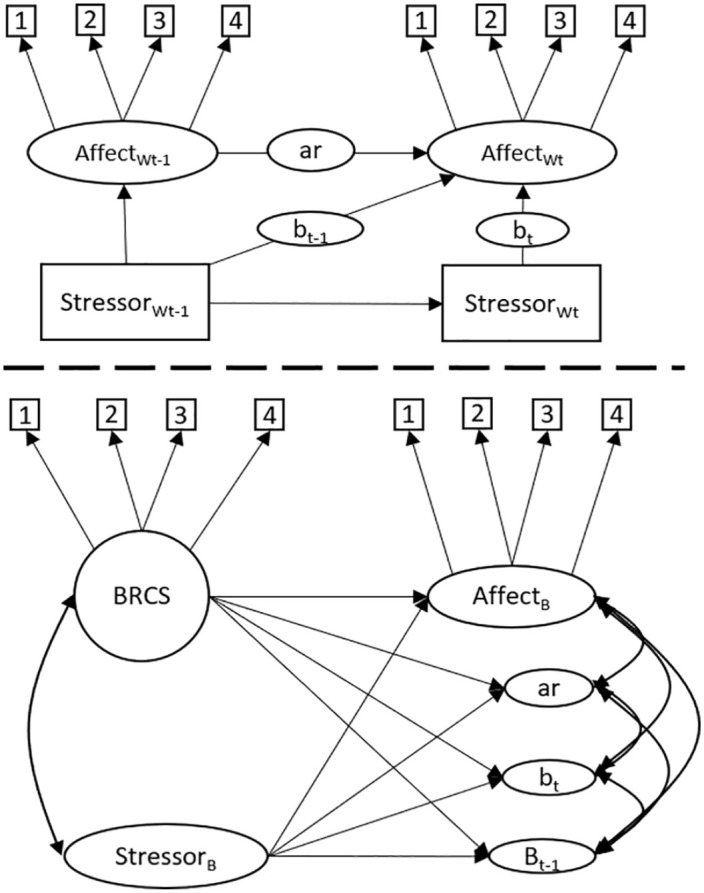
The Hypothesized Dynamic Structural Equation Model.

Figure 2 depicts the proposed DSEM that can be used to analyze to what degree individual differences in affect *reactivity* to stressors and *recovery* from stressors are explained by the BRCS questionnaire. Above the dashed line is the within-person level of the model, denoted by the subscript *W*. Square boxes represent manifest items, where the subscript *t* denotes time, and *t*−1 denotes the previous time point. Arrows represent regressions and factor loadings. Affect is measured by four indicators and is regressed on affect at *t*−1, and stressful events at *t* and *t*−1. Stressor is a sum score of six dichotomous items and is regressed on stressor at *t*−1. Circles on regression arrows indicate that each participant gets their own regression parameters by combining data across all time points, where *ar* represents the autoregressive slope for affect, *b_t_* represents affect *reactivity* to stressors at the same time point, while *b_t_*_−1_ represents *recovery* from stressors at the previous time point. On the between level, below the dashed line, these latent variables are included in a structural equation model that is interpreted in the same way as a common between-person analysis. For example, by regressing *b_t_* on BRCS, we are analyzing how much of the variance in affective reactivity between respondents is explained by their response to the BRCS questionnaire. We expected that BRCS would explain variance in *b_t_* and *b_t_*_−1_ with a moderate to large effect size. Affect_B_ and Stressor_B_ represent the respondents’ mean level of affect and stressors across time, respectively. In the present study, we ran two models, one for PA, and one for NA. Model convergence was evaluated by checking that the potential scale reduction (PSR) values were consistently below 1.05 (Asparouhov & Muthen, 2023), and by inspecting posterior trace plots for all parameters.

### Open Practice Statement

The data and Mplus syntax for the study are publicly accessible at https://osf.io/hmysc/. The study was not preregistered.

## Results

### IRT Analysis of the BRCS Measure

Exploratory factor analysis was used to test the assumption of unidimensionality of the BRCS. All items loaded on a single factor (geomin rotated loadings .467–.596). The eigenvalue for the first factor was 1.886, and the second .837, giving a ratio of 2.25. To evaluate the assumption of local independence, we ran a confirmatory factor analysis where the four items loaded on a single factor. The model had acceptable fit: χ^2^(2) = 4.1, *p* = .12, *n* = 177; root mean square error of approximation (RMSEA) = .078; comparative fit index (CFI) = .970; standardized root mean square residual (SRMR) = .03. Absolute values for residual correlations were less than .09, and modification indices did not suggest correlating any items, indicating there were no local dependencies.

Next, we compared the GPCM model to a 1PL model by constraining the discriminations to be equal across all items. Results of the likelihood ratio chi-square difference test, χ^2^(3) = .691, *p* = .88, and comparison of Akaike information criterion (AIC) (AIC_full_ = 1653.062, AIC_constrained_ = 1648.264) and Bayesian information criterion (BIC) (BIC_full_ = 1713.409, BIC_constrained_ = 1698.976) indicated that the constrained model did not fit worse, and thus that the discrimination factors can be held equal across items.

**Figure 3.**
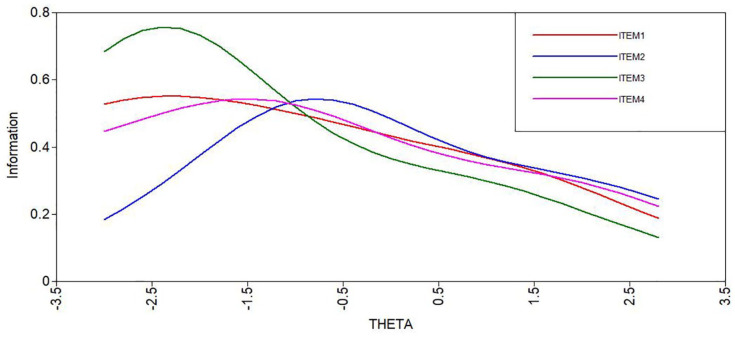
Item Information Curves as a Function of the Latent Trait Resilience (THETA).

**Figure 4:**
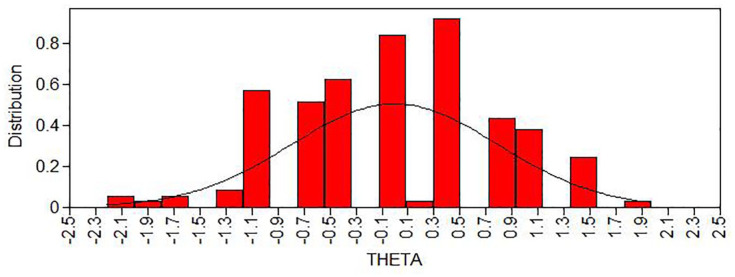
Estimated Scores on the Latent Variable Resilience (THETA). *Note. n* = 177, *M* = .001, standard deviation = .791, skewness = –.039, kurtosis = –.135.

The item discrimination was moderate (.945). The item locations were mostly on the lower side (item 1 = −1.37, item 2 = .11, item 3 = −1.65, item 4 = −1.22); see also Figure 3 for item information curves. The results indicate that the BRCS provides more information about respondents who have low scores on the latent trait resilience but are less able to discriminate among highly resilient individuals. The estimated scores on the latent resilience factor were normally distributed (Figure 4). Taken together, this shows that the BRCS measured resilience with quite low reliability in our sample, which leads to increased standard errors.

### Descriptives

Between-person estimated means for individual items are presented in Table 1. Notably, for the momentary assessment items (i.e., the items for affect and stressors), the means in the table describe an average (between individuals) of averages (within individuals), and thus give a first picture of the data, but does not account for the clustering of experiences.

**Table 1.** Descriptive Statistics for Study Variables at the Between-Person Level.

	Morning		Afternoon		Minimum	Maximum
Variable	*M*	*SD*	*M*	*SD*		
Positive affect						
1. Work feels meaningful	3.86	.58	3.76	.59	1	5
2. Work feels fun	3.66	.64	3.54	.60	1	5
3. Work feels manageable	3.86	.65	3.73	.67	1	5
4. I feel appreciated	3.42	.71	3.33	.64	1	5
Negative affect						
1. I feel stressed	2.80	.86	2.96	.92	1	5
2. I feel tired**	2.89	.96	3.17	.96	1	5
Stressors						
1. I’ve been able to work at my own pace (r)**	.64	.31	.53	.35	0	1
2. I’ve had time for breaks (r)	.19	.28	.19	.27	0	1
3. There has been a lot going on***	.31	.29	.43	.32	0	1
4. I’ve been given unexpected tasks	.10	.20	.13	.23	0	1
5. I’ve been badly treated	.03	.12	.02	.08	0	1
6. I’ve seen someone being badly treated***	.04	.12	.10	.18	0	1
Brief Resilient Coping Scale	*M*	*SD*			Minimum	Maximum
1. I actively look for ways to replace the losses I encounter in life	3.74	.83			1	5
2. I believe that I can grow in positive ways by dealing with difficult situations	3.52	.86			2	5
3. I look for creative ways to alter difficult situations	4.01	.79			1	5
4. Regardless of what happens to me, I believe I can control my reaction to it	3.65	.83			1	5

*Note.* (r) = reversed item. Stars denote statistically significant mean differences between morning and afternoon measurement.

* *p <* .05. **p < .01. ***p < .001.

### Momentary Assessment Data

To test to what extent the variation is located at the between-person versus the within-person level, ICCs were calculated. The ICCs for stress (.29), NA (.51), and PA (.43), all showed that a noticeable part of each variable varied within persons and thus suggested a multilevel framework.

### Main Analyses

When including the parameter *b_t_*_−1_ between affect at timepoint *t* and stressor at time *t*−1, the models either did not converge or displayed unstable trace plots. In addition, PSR values were not reliably low but fluctuated around 1.2 after tens of thousands of iterations. Consequently, this parameter was left out of the models.

**Figure 5:**
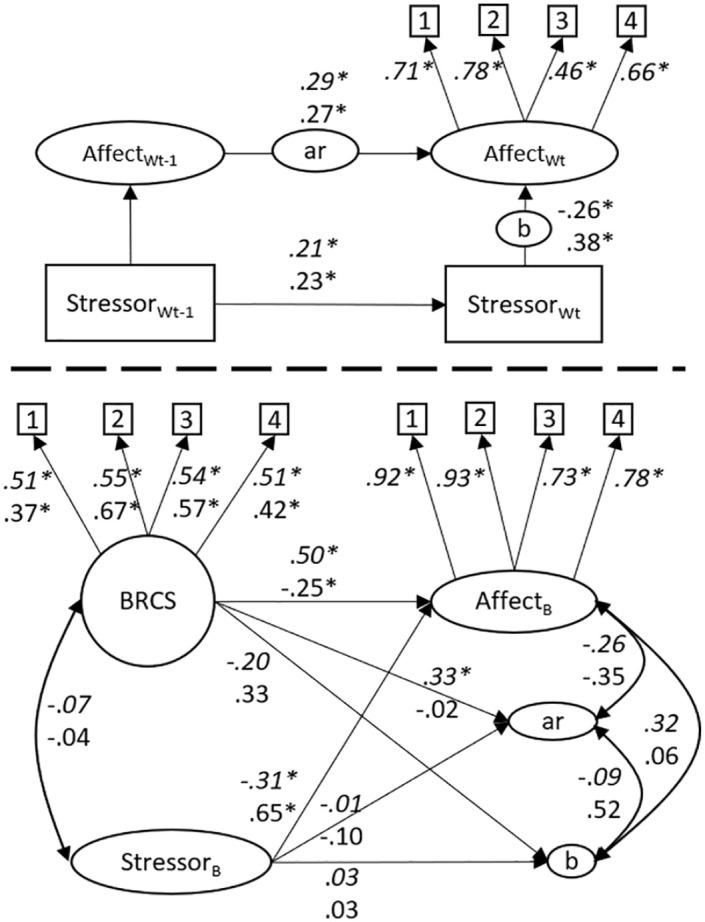
Dynamic Structural Equation Models of Resilience as Measured by Affective Reactivity to Stressors and by the Brief Resilient Coping Scale. *Note.* Cursive numbers (above) depict standardized parameter estimates from the model of positive affect, and non-cursive numbers (below) depict estimates from the model of negative affect. Positive affect is a latent factor measured by four indicators, while negative affect is a sum score of two items. BRCS = Brief Resilient Coping Scale. The main parameters of interest for the hypothesis are the regression parameters between b and BRCS. *Significant parameter.

Results from the main analyses are depicted in Figure 5. As expected, on the within level, stressors during the last few hours predicted current affect in the expected direction (i.e., stressors reduced positive affect and increased NA).

On the between-level, contrary to our hypothesis, individual differences in affective reactivity to stressors were not predicted by the BRCS measure (β_positive affect_ [95% CI] = −.20, [−.51, .11]; β_negative affect_ = .33, [−.07, .69]). Reactivity was not related to participants’ mean levels of affect nor stress.

The BRCS did, however, predict the respondents’ mean levels of PA and NA, so the respondents who score higher on BRCS in general report higher levels of PA and lower levels of NA. In a similar way, the mean level of stressor did not predict reactivity but did predict mean levels of affect, so higher mean levels of stressful events predicted lower levels of PA and higher levels of NA. Parameter estimates are presented in Tables 2 and 3. R-square estimates are presented in Table 4.

**Table 2.** Standardized Parameter Estimates for Model of Positive Affect.

Parameter	Standardized estimate	Posterior *SD*	*p*	95% CI	
				*LL*	*UL*
*Within-level*					
Affect by *1. work feels meaningful*	.71*	.02	.00	.67	.73
Affect by *2. work feels fun*	.78*	.01	.00	.75	.80
Affect by *3. work feels manageable*	.46*	.03	.00	.41	.51
Affect by *4. I feel appreciated*	.66*	.02	.00	.62	.69
Affect auto-regression (ar)	.29*	.04	.00	.22	.36
Affect on stressor (b_t_)	−.26*	.03	.00	−.31	−.19
Stressor auto-regression	.21*	.04	.00	.13	.20
*Between-level*					
Affect by *1. work feels meaningful*	.92*	.03	.00	.85	.96
Affect by *2. work feels fun*	.93*	.03	.00	.87	.97
Affect by *3. work feels manageable*	.73*	.05	.00	.62	.81
Affect by *4. I feel appreciated*	.78*	.04	.00	.68	.85
BRCS by *1. Look for ways to replace losses*	.51*	.09	.00	.29	.66
BRCS by *2. Grow in positive ways*	.55*	.08	.00	.37	.69
BRCS by *3. Look for creative ways*	.54*	.08	.00	.37	.68
BRCS by *4. Control my reaction*	.51*	.08	.00	.34	.65
Affect on BRCS	.50*	.10	.00	.31	.68
ar on BRCS	.33*	.16	.02	.00	.63
b on BRCS	−.20	.16	.10	−.51	.11
Affect on stressor	−.31*	.09	.00	−.49	−.14
ar on stressor	−.01	.15	.48	−.31	.27
b on stressor	.03	.15	.43	−.27	.32
Affect with ar	−.26	.21	.12	−.66	.16
Affect with b	.32	.19	.04	−.04	.70
ar with b	−.09	.26	.36	−.66	.37
BRCS with stressor	−.07	.11	.25	−.29	.15

*Note.* “by” denotes a factor loading, “on” denotes a regression coefficient, “with” denotes a correlation. CI = confidence interval; LL = lower limit; UL = upper limit; BRCS = Brief Resilient Coping Scale.

* Significant parameter.

**Table 3.** Parameter Estimates for Model With Negative Affect.

Parameter	Standardized estimate	Posterior *SD*	*p*	95% CI	
				*LL*	*UL*
*Within-level*					
Affect auto-regression (ar)	.27*	.04	.00	.21	.34
Affect on stressor (b)	.38*	.02	.00	.34	.42
Stressor auto-regression	.23*	.03	.00	.16	.30
*Between-level*					
BRCS by *1. Look for ways to replace losses*	.37*	.13	.00	.08	.57
BRCS by *2. Grow in positive ways*	.66*	.09	.00	.48	.84
BRCS by *3. Look for creative ways*	.57*	.08	.00	.39	.72
BRCS by *4. Control my reaction*	.42*	.09	.00	.23	.59
Affect on BRCS	−.25*	.08	.00	−.41	−.09
ar on BRCS	−.02	.21	.47	−.41	.40
b on BRCS	.33	.19	.05	−.07	.69
Affect on stressor	.65*	.06	.00	.53	.75
ar on stressor	−.10	.18	.28	−.44	.27
b on stressor	.03	.19	.44	−.34	.41
Affect with ar	−.35	.21	.05	−.75	.07
Affect with b	.06	.22	.40	−.33	.50
ar with b	.52	.30	.07	−.22	.94
BRCS with stressor	.04	.11	.35	−.26	.18

*Note.* “by” denotes a factor loading, “on” denotes a regression coefficient, “with” denotes a correlation. CI = confidence interval; LL = lower limit; UL = upper limit; BRCS = Brief Resilient Coping Scale.

* Significant parameter.

**Table 4:** R-Square Estimates.

	Negative affect				Positive affect			
			95% CI				95% CI	
Variable	Estimate	*SD*	LL	UL	Estimate	*SD*	LL	UL
*Within-level R-square averaged across clusters*								
Stressor	.05	.02	.03	.09	.05	.02	.02	.08
Negative affect sum	.31	.02	.26	.36				
Work feels meaningful					.51	.02	.45	.54
Work feels fun					.60	.02	.56	.65
Work feels manageable					.22	.02	.18	.27
I feel appreciated					.44	.03	.40	.49
*Between level*								
1. I actively look for ways to replace the losses I encounter in life	.14	.09	.01	.32	.26	.09	.09	.44
2. I believe that I can grow in positive ways by dealing with difficult situations	.44	.12	.23	.70	.30	.09	.14	.48
3. I look for creative ways to alter difficult situations	.32	.10	.16	.52	.29	.09	.13	.47
4. Regardless of what happens to me, I believe I can control my reaction to it	.17	.07	.05	.34	.26	.08	.11	.42
Negative affect sum	.50	.07	.36	.64				
Work feels meaningful					.84	.05	.73	.92
Work feels fun					.87	.05	.76	.95
Work feels manageable					.53	.07	.38	.66
I feel appreciated					.60	.07	.46	.72

*Note.* CI = confidence interval; LL = lower limit, UL = upper limit.

### Evaluation of Models

To evaluate robustness of the results, we more than doubled the number of iterations. PSR values for both models and posterior trace plots for all parameters were satisfactory. To evaluate the sensitivity of the models, we ran models using only single daily measures as well as models based on sum scores of the BRCS scale and PA (rather than latent variables). The interpretations of the main results were the same in these analyses, leading us to conclude that the models are stable.

## Discussion

In their original paper where the BRCS was introduced, Sinclair and Wallston (2004) discuss different conceptualizations of resilience, and state that certain resilience-related personality traits and protective environmental factors provide a substrate for developing and refining coping skills that are used when facing adversity. Previous empirical studies have found associations between big-five personality traits and affective reactivity to stressful events (Komulainen et al., 2014; Leger et al., 2021). The present study was the first to compare a questionnaire-based retrospective self-report measure of resilience to indirectly measured in-the-moment resilience processes. Our results corroborate that the BRCS captures some individual differences, as those who scored higher on the BRCS also reported higher mean levels of PA and lower levels of NA. However, contrary to our hypothesis, the questionnaire failed to predict individual differences in affective reactivity to stressors. Even if the parameters in question had been statistically significant, which they were not, the effect sizes were smaller than expected.

One possible interpretation of the results is that the theoretical assumption that resilience-related personality traits trickle down into resilient reactivity in the face of adversity is not correct, at least not for the operationalization of resilience as affective reactivity, and that the BRCS measure instead captures other stable individual differences such as general mood, which might be relevant in a resilience-context. Another explanation for this null result is that the BRCS measure has limited reliability. Indeed, as the IRT analyses indicate, the items are rather imprecise and do not cover the entire spectrum of the latent variable, notably providing little information for positive scores of resilience. This means the measure includes errors that can increase standard errors and possibly bias estimates. As such, our findings are limited to this specific measure of trait resilience, and future studies could include other questionnaire-based measures of resilience.

Yet another interpretation of these findings is that the trait-based and the process-based operationalizations capture different aspects of resilience as it unfolds over time. We can imagine resilience as a process with four stages: (a) you face adversity of some degree of severity, (b) you are emotionally and cognitively affected by the adversity, (c) you decide how to behave, or deal with, or react to the situation, which in turn (d) leads to some form of outcome. In this model, the affective reactivity operationalization is involved in step 2, while the BRCS, on the other hand, can be understood as focusing on cognitive and behavioral coping skills in step 3. Both steps 2 and 3 affect the outcome in step 4. Indeed, prospective studies have found affective reactivity to predict outcomes such as marital satisfaction and heart rate variability (Ong et al., 2020) and allostatic load (Piazza et al., 2019), and the BRCS to predict outcomes such as post-traumatic stress disorder (Sinclair et al., 2020) and depression (Gullo et al., 2021). Nevertheless, steps 2 and 3 are not necessarily associated with each other. Seen from this perspective, our results can be seen to implicate that the two operationalizations capture different aspects of the multi-faceted concept of resilience.

Two important differences between the operationalization are the degree to which they rely on retrospection and self-report. Regarding retrospection, studies have found that everyday experiences reported by means of immediate introspection and long-term evaluations of experiences are not necessarily in perfect correspondence (Kahneman & Riis, 2005). For example, the validity of the BRCS relies on the participant’s ability to accurately retrieve experiences from their past and correctly integrate them into a rating of how they usually deal with challenges. In contrast, for process-based operationalization, affect is measured in the immediate experience, while stressful events happened within the last few hours, thus relying far less on retrospection. Another potential problem is that asking people to rate themselves on how good they are at dealing with problems is related to desirability biases (Krumpal, 2013). We might want to uphold a presentation of competence toward ourselves and others, which can lead us to overestimate our resilience. On this point, the affective reactivity measure is again favorable since it does not involve asking about resilience directly. Rather, it is based on questions of the occurrence of stressors and current affect, which is arguably more neutral and objective, and thus not as sensitive to desirability biases.

The present study has some limitations that need to be considered. First, the participants represent a highly educated group of professionals within a language minority in Finland. In addition, the data was collected during the Covid pandemic. Further, it is conceivable that participants with very high levels of stress opted not to participate, due to an already high workload. These factors might limit the generalizability of the findings. To generalize the findings, future studies need to include other samples and contexts. In doing so, it is necessary to adapt the in-the-moment operationalization, since different types of stressors are relevant in different contexts. For example, comparing occupational contexts, different things might be considered relevant stressors in a customer-facing retail job compared to nursing in a hospital ward. Second, while the sample size would be appropriate for regular between-person analyses, it might have been insufficient for the complex DSEM analyses. As noted by Schultzberg and Muthén (2018), in terms of power assumptions for DSEMs, a large *N* can compensate for a small number of time points better than a large number of time points can compensate for a small *N*. However, for a model with both an auto-regressive effect and a within-person predictor, a larger T than 16 would be preferable. This might explain why the models did not converge in a satisfactory manner when including the *b_t_*_−1_ parameter. Another reason could be that as we had two measurement occasions per day, we only have observations of one cross-lagged effect (between morning and afternoon), whereas for the within-time effects, we have two observations per day. Due to how Mplus handles time to make measurements equidistant, each day has eight measurement occasions, with missing values added for six occasions per day. With only a single observation per day for the cross-lagged effect, the theoretical maximum covariance coverage for that parameter is .125, which in practice (with missing data) might mean we had insufficient data to model this parameter.

In conclusion, the results of the present study call into question the validity of measuring resilience using the BRCS. Our findings further suggest that researchers planning studies on resilience need to decide on whether they want to study the intraindividual processes of resilience, and, if so, should not measure resilience by means of a between-person level questionnaire. Further research is needed to evaluate different trait and process operationalizations of resilience, and how they correspond to each other and outcomes of interest.

## References

[bibr1-10731911241234220] Anderson S. R. Miller R. B. (2020). Improving measurement in couple and family therapy: An item response theory primer. Journal of Marital and Family Therapy, 46(4), 603–619. 10.1111/jmft.12448 32776620

[bibr2-10731911241234220] Asparouhov T. Muthen B. (2023). Bayesian analysis using Mplus: Technical implementation. http://www.statmodel.com/download/Bayes2.pdf

[bibr3-10731911241234220] Beltman S. (2020). Understanding and examining teacher resilience from multiple perspectives. In Mansfield C. F. (Ed.), Cultivating teacher resilience: International approaches, applications and impact (pp. 11–26). Singapore: Springer. 10.1007/978-981-15-5963-1

[bibr4-10731911241234220] Beltman S. Mansfield C. Price A. (2011). Thriving not just surviving: A review of research on teacher resilience. Educational Research Review, 6(3), 185–207. 10.1016/j.edurev.2011.09.001

[bibr5-10731911241234220] Eisele G. Vachon H. Lafit G. Kuppens P. Houben M. Myin-Germeys I. Viechtbauer W. (2022). The effects of sampling frequency and questionnaire length on perceived burden, compliance, and careless responding in experience sampling data in a student population. Assessment, 29(2), 136–151. 32909448 10.1177/1073191120957102

[bibr6-10731911241234220] Finnish National Board on Research Integrity TENK. (2019). The ethical principles of research with human participants and ethical review in the human sciences in Finland (3/2019).

[bibr7-10731911241234220] Gullo S. Misici I. Teti A. Liuzzi M. Chiara E. (2021). Going through the lockdown: A longitudinal study on the psychological consequences of the coronavirus pandemic. Research in Psychotherapy: Psychopathology, Process and Outcome, 23(3). 10.4081/ripppo.2020.494 PMC7875066 33585300

[bibr8-10731911241234220] Hamaker E. L. Asparouhov T. Brose A. Schmiedek F. Muthén B. (2018). At the frontiers of modeling intensive longitudinal data: Dynamic structural equation models for the affective measurements from the COGITO study. Multivariate Behavioral Research, 53(6), 820–841. 10.1080/00273171.2018.1446819 29624092

[bibr9-10731911241234220] Hamaker E. L. Wichers M. (2017). No time like the present: Discovering the hidden dynamics in intensive longitudinal data. Current Directions in Psychological Science, 26(1), 10–15. https://doi.org/10.1177/0963721416666518/

[bibr10-10731911241234220] Howard S. Johnson B. (2004). Resilient teachers: Resisting stress and burnout. Social Psychology of Education, 7(4), 399–420. 10.1007/s11218-004-0975-0

[bibr11-10731911241234220] Kahneman D. Riis J. (2005). Living, and thinking about it: Two perspectives on life. In Huppert F. A. Baylis N. Keverne B. (Eds.), The science of well-being (pp. 285–304). Oxford University Press.

[bibr12-10731911241234220] Kalisch R. Baker D. G. Basten U. Boks M. P. Bonanno G. A. Brummelman E. Chmitorz A. Fernàndez G. Fiebach C. J. Galatzer-Levy I. Geuze E. Groppa S. Helmreich I. Hendler T. Hermans E. J. Jovanovic T. Kubiak T. Lieb K. Lutz B. Kleim B. (2017). The resilience framework as a strategy to combat stress-related disorders. Nature Human Behaviour, 1(11), 784–790. 10.1038/s41562-017-0200-8 31024125

[bibr13-10731911241234220] Komulainen E. Meskanen K. Lipsanen J. Lahti J. M. Jylhä P. Melartin T. Wichers M. Isometsä E. Ekelund J. (2014). The effect of personality on daily life emotional processes. PLoS ONE, 9(10), Article e110907. 10.1371/journal.pone.0110907 PMC4208812 25343494

[bibr14-10731911241234220] Krumpal I. (2013). Determinants of social desirability bias in sensitive surveys: A literature review. Quality & Quantity, 47(4), 2025–2047. 10.1007/s11135-011-9640-9

[bibr15-10731911241234220] Laurenceau J-P. Bolger N. (2021, June 21-25). Analyzing intensive longitudinal data. [Lecture notes]. CenterStat.

[bibr16-10731911241234220] Leger K. A. Turiano N. A. Bowling W. Burris J. L. Almeida D. M. (2021). Personality traits predict long-term physical health via affect reactivity to daily stressors. Psychological Science, 32(5), 755–765. 10.1177/0956797620980738 33882261 PMC8258308

[bibr17-10731911241234220] Luthar S. S. Cicchetti D. Becker B. (2000). The construct of resilience: A critical evaluation and guidelines for future work. Child Development, 71(3), 543–562. 10.1111/1467-8624.00164 10953923 PMC1885202

[bibr18-10731911241234220] Malloy W. W. Allen T. (2007). Teacher retention in a teacher resiliency-building rural school. The Rural Educator, 28(2), 19–27. 10.35608/ruraled.v28i2.482

[bibr19-10731911241234220] Mansfield C. F. Beltman S. Broadley T. Weatherby-Fell N. (2016). Building resilience in teacher education: An evidenced informed framework. Teaching and Teacher Education, 54, 77–87. 10.1016/j.tate.2015.11.016

[bibr20-10731911241234220] Muraki E. (1997). A generalized partial credit model. In van der Linden W. J. Hambleton R. K. (Eds.), Handbook of modern item response theory (pp. 153–164). Springer. 10.1007/978-1-4757-2691-6_9

[bibr21-10731911241234220] Myin-Germeys I. Kuppens P. (Eds.). (2021). The open handbook of experience sampling methodology: A step-by-step guide to designing, conducting, and analyzing ESM studies. The Center for Research on Experience Sampling and Ambulatory Methods Leuven (REAL).

[bibr22-10731911241234220] Ong A. D. Gardner S. Urganci B. Gunaydin G. Selcuk E. (2020). Affective reactivity, resting heart rate variability, and marital quality: A 10-year longitudinal study of U.S. adults. Journal of Family Psychology, 34(3), 375–382. 10.1037/fam0000591 31464453 PMC7048653

[bibr23-10731911241234220] Ong A. D. Leger K. A. (2022). Advancing the study of resilience to daily stressors. Perspectives on Psychological Science, 17(6), 1591–1603. 35748196 10.1177/17456916211071092 PMC10122438

[bibr24-10731911241234220] Piazza J. R. Stawski R. S. Sheffler J. L. (2019). Age, daily stress processes, and allostatic load: A longitudinal study. Journal of Aging and Health, 31(9), 1671–1691. 10.1177/0898264318788493 30019595 PMC6312754

[bibr25-10731911241234220] Schultzberg M. Muthén B. (2018). Number of subjects and time points needed for multilevel time-series analysis: A simulation study of dynamic structural equation modeling. Structural Equation Modeling, 25(4), 495–515. 10.1080/10705511.2017.1392862

[bibr26-10731911241234220] Sinclair V. G. Adams S. M. Dietrich M. (2020). Associations between changes in resilient coping and posttraumatic stress disorder symptoms. Research in Nursing & Health, 43(3), 255–262. 10.1002/nur.22014 32067237

[bibr27-10731911241234220] Sinclair V. G. Wallston K. A. (2004). The development and psychometric evaluation of the Brief Resilient Coping Scale. Assessment, 11(1), 94–101. 10.1177/1073191103258144 14994958

[bibr28-10731911241234220] Su P. Yi J. Chen X. Xiao Y. (2023). Visual analysis of psychological resilience research based on Web of Science database. Psychology Research and Behavior Management, 16, 465–481. 10.2147/PRBM.S394693 36846313 PMC9948642

[bibr29-10731911241234220] Watson D. Clark C. L. Tellegen A. (1988). Development and validation of brief measures of positive and negative affect: the PANAS scales. Journal of Personality and Social Psychology, 54(6):1063–1070. 10.1037/0022-3514.54.6.1063 3397865

